# Local Delivery of Azithromycin Nanoformulation Attenuated Acute Lung Injury in Mice

**DOI:** 10.3390/molecules27238293

**Published:** 2022-11-28

**Authors:** Mohsen G. Alrashedi, Ahmed Shaker Ali, Osama Abdelhakim Ahmed, Ibrahim M. Ibrahim

**Affiliations:** 1 Department of Pharmacology, Faculty of Medicine, King Abdulaziz University, Jeddah 21589, Saudi Arabia; 2 Ministry of Health, Riyadh 12628, Saudi Arabia; 3 Department of Pharmaceutics, Faculty of Pharmacy, Assiut University, Assiut 71515, Egypt; 4 Department of Pharmaceutics, Faculty of Pharmacy, King Abdulaziz University, Jeddah 21589, Saudi Arabia

**Keywords:** antiviral, nanostructured lipid carrier, bleomycin, lung targeting, intratracheal delivery

## Abstract

Humanity has suffered from the coronavirus disease 2019 (COVID-19) pandemic over the past two years, which has left behind millions of deaths. Azithromycin (AZ), an antibiotic used for the treatment of several bacterial infections, has shown antiviral activity against severe acute respiratory syndrome coronavirus 2 (SARS-CoV-2) as well as against the dengue, Zika, Ebola, and influenza viruses. Additionally, AZ has shown beneficial effects in non-infective diseases such as cystic fibrosis and bronchiectasis. However, the systemic use of AZ in several diseases showed low efficacy and potential cardiac toxicity. The application of nanotechnology to formulate a lung delivery system of AZ could prove to be one of the solutions to overcome these drawbacks. Therefore, we aimed to evaluate the attenuation of acute lung injury in mice via the local delivery of an AZ nanoformulation. The hot emulsification–ultrasonication method was used to prepare nanostructured lipid carrier of AZ (AZ-NLC) pulmonary delivery systems. The developed formulation was evaluated and characterized in vitro and in vivo. The efficacy of the prepared formulation was tested in the bleomycin (BLM) -mice model for acute lung injury. AZ-NLC was given by the intratracheal (IT) route for 6 days at a dose of about one-eighth oral dose of AZ suspension. Samples of lung tissues were taken at the end of the experiment for immunological and histological assessments. AZ-NLC showed an average particle size of 453 nm, polydispersity index of 0.228 ± 0.07, zeta potential of −30 ± 0.21 mV, and a sustained release pattern after the initial 50% drug release within the first 2 h. BLM successfully induced a marked increase in pro-inflammatory markers and also induced histological changes in pulmonary tissues. All these alterations were significantly reversed by the concomitant administration of AZ-NLC (IT). Pulmonary delivery of AZ-NLC offered delivery of the drug locally to lung tissues. Its attenuation of lung tissue inflammation and histological injury induced by bleomycin was likely through the downregulation of the p53 gene and the modulation of Bcl-2 expression. This novel strategy could eventually improve the effectiveness and diminish the adverse drug reactions of AZ. Lung delivery could be a promising treatment for acute lung injury regardless of its cause. However, further work is needed to explore the stability of the formulation, its pharmacokinetics, and its safety.

## 1. Introduction

Coronavirus disease 2019 (COVID-19) is a global pandemic caused by the highly infectious respiratory virus severe acute respiratory syndrome coronavirus 2 (SARS-CoV-2). It caused millions of deaths and crushing effects on the economy [1,2,3]. More than 633 million cases had been confirmed as of 16 November 2022, with more than 6 million verified deaths [4]. Azithromycin (AZ) was one of the drugs repurposed for the management of COVID-19. An in vitro study demonstrated the effect of AZ as a monotherapy against SARS-CoV-2, while other studies found that it potentiates the in vitro antiviral activity of hydroxychloroquine (HCQ) [5]. In an in vitro study, complete inhibition of SARS replication was demonstrated after an incubation period of 60 h with the combination of HCQ 2 µM and AZ 10 µM [6]. However, several systematic reviews and metanalyses concluded that AZ administration by an oral route in safe doses did not result in a superior clinical improvement in COVID-19 patients [7,8]. The antiviral activity of AZ has also been shown to be effective against other viruses including the dengue, Zika, Ebola, and influenza viruses [9]. Other lung conditions associated with inflammation include cystic fibrosis where AZ showed an improvement in respiratory function after six months of therapy [10]. Furthermore, AZ has been shown to prevent exacerbations of chronic obstructive pulmonary disease [11]. In addition, randomized controlled trials concluded that AZ was effective in preventing bronchiolitis obliterans syndrome after lung transplantation [12] and ventilator-induced lung injury by decreasing cytokines’ production [13]. 

However, systemic administration of AZ has been associated with many adverse effects including cardiotoxicity [14,15,16,17]. The potentially serious side effect and low efficacy of the conventional dosage forms of AZ limited its utilization in patients with COVID-19. In addition, there was a risk of the emergence of macrolide resistance with the prolonged administration for other chronic lung conditions. This necessitates the establishment of effective approaches to surpass the limitations of conventional dosage forms.

From this standpoint, interest was built in studying AZ and its role in treating acute lung injury caused by the above-mentioned conditions, and in developing methods for its use via inhalation. The inhalation route of AZ is promising; for example, the adoption of the spray drying technique for dry powder formulations of AZ was successfully achieved with good inhaler characteristics. An in vivo study on mice confirmed that intratracheal (IT) administration of these formulations achieves higher concentrations in the lung tissues compared to administration via intravenous administration. The drug targeting index of the IT route was 486.2 [18]. Another inhaled formulation of AZ was recently developed by co-spraying with L-Lucine [19]. Pure AZ dry powder inhalers were also developed and showed promising inhalation characteristics [20]. In vivo experiments using inhaled macrolides in rats showed high concentrations and areas under the curve that were enough for antimicrobial therapy in alveolar macrophages and epithelial lining fluid without lung damage [21].

Pulmonary drug delivery has many merits, for example, fewer systemic toxicities, higher bioavailability, and rapid action of the drugs. These features make lung delivery of the most anti-infective drugs an optimal route for the treatment of pulmonary disorders [22]. In line with that, nanotechnology has received a lot of interest for its utility in the management of viral infections including COVID-19 [23,24,25]. Nanocarrier delivery systems have unique advantages, such as the increased bioavailability and ability to entrap drugs with different physicochemical properties [26]. Nanostructured lipid carriers (NLC) mainly consist of biocompatible lipids, surfactants, and co-surfactants. NLC has characteristics that make it a favorable drug delivery system, i.e., a simple manufacturing process, biocompatibility, scale-up feasibility, non-toxicity, and increased loading capacity and stability [27]. The current study aimed to develop AZ by using NLC as a nanoformulation given by inhalation to attenuate acute lung injury in mice.

## 2. Results

### 2.1. In Vitro Characterization of AZ-NLC

The mean measured particle size of AZ-NLC was 453 ± 26 nm. The zeta potential value was −30 ± 0.21 mV and the PDI was 0.228 ± 0.07.

**Figure 1:**
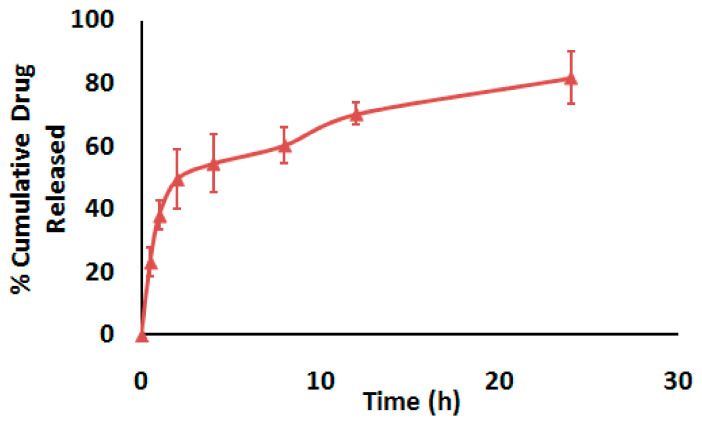
In vitro drug release from AZ-NLC at 37 °C. Data are presented as mean ± SD, n = 3.

In vitro release profiles of AZ-NLC are graphically illustrated in Figure 1. It was evident that AZ-NLC exhibited an initial enhanced drug release stage within the first 2 h (around 50%) followed by a sustained release pattern.

### 2.2. In Vivo Pro-Inflammatory Markers Evaluation

**Figure 2:**
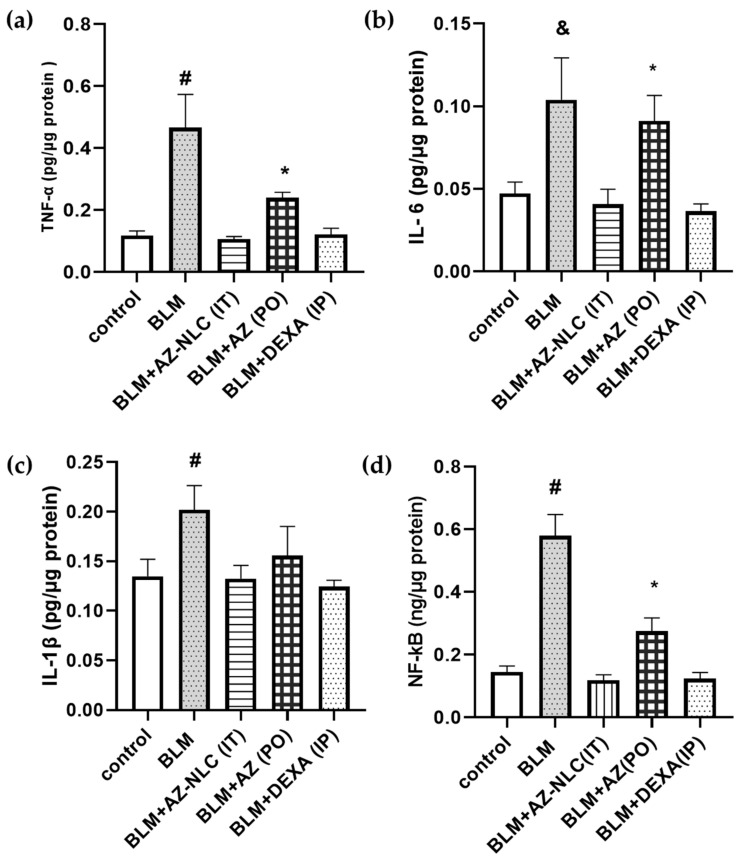
Effect of AZ formulations (AZ-NLC IT and AZ PO) on (**a**) TNF-α, (**b**) IL-6, (**c**) IL-1β, and (**d**) NF-kB concentrations in lung tissues. Mice were administered BLM (2.5 mg/kg) as a single dose followed by six doses once daily of either AZ-NLC IT (11 mg/kg), AZ (88 mg/kg) PO, or DEXA (5 mg/kg) IP for 6 successive days. # Significant (*p* < 0.05) versus all groups. & Significant (*p* < 0.05) versus all groups except the oral group. * Significant (*p* < 0.05) versus the control group and AZ (IT) group and DEXA group. AZ: azithromycin; BLM: bleomycin; DEXA: dexamethasone; IT: intratracheal; PO: oral; IP: intraperitoneal; NLC: nanostructured lipid carriers.

The effect of various AZ formulations on pro-inflammatory markers in lung tissue homogenate after repeated administration for 6 days in mice with BLM-induced acute lung injury is shown in Figure 2. BLM-induced acute lung injury was associated with a significant increase in the mean concentration of TNF-α, IL-6, IL-1B, and NF-kB in lung tissues compared to that in the control (*p* < 0.05). Treatment with AZ-NLC nanoformulation IT significantly attenuated the BLM-induced increase in all tested parameters. The mean TNF-α, IL-6, and NF-kB in lung tissues of mice that received IT AZ-NLC at a low dose (11 mg/kg) were significantly lower than that after an oral dose of the AZ suspension.

### 2.3. Histopathological Changes

**Figure 3:**
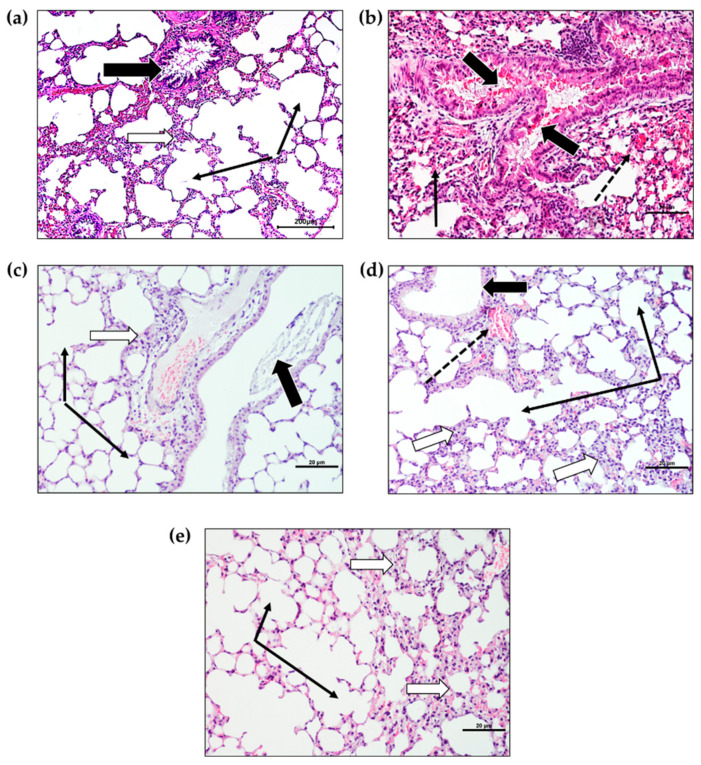
Sections of lung tissues of BLM-induced acute lung injury after administration of AZ-NLC IT compared with other groups (H&E stain, ×100 Bar =200 µm). (**a**) Control: normal alveoli with no inflammatory cells (thin black arrows) separated by thin interalveolar septa. Bronchiole showed a normal appearance (white arrow). (**b**) BLM: bronchioles (thick black arrows) lumina contained cellular desquamation and hemorrhage (white stars), collapsed alveoli, or filled with inflammatory cells (thin black arrow) and capillary congestion (dotted arrows). (**c**) BLM + AZ (IT) bronchioles were similar to the control but showed few foci with interalveolar inflammatory infiltrate. (**d**) BLM + AZ (PO) showed moderate improvement with most alveoli opened but with foci of interalveolar septa thickening. (**e**) BLM + DEXA (IP): the alveoli were free of inflammation to infiltrate and showed thin interalveolar septa with few connective tissue cells.

The lung tissues of mice showed all the features of healthy lungs with the administered vehicle (control). The bronchioles were normal with an intact epithelial lining and empty lumen. Most alveoli showed features such as an opened lumina, being free of any inflammatory cells, separated by thin interalveolar septa, and thin-walled non-congested capillaries. BLM administration caused marked histological changes such as epithelial lining degeneration with desquamation and hemorrhage. The alveolar structure exhibited marked disorganization. The interalveolar septa were thickened with marked infiltration of inflammatory cells (Figure 3). The lungs of mice receiving daily IT AZ-NLC showed normalization in most histological changes induced by BLM. The lung structures appeared similar to those after oral administration of AZ suspension in a higher dose of IP DEXA.

### 2.4. Immunohistochemistry Findings

**Figure 4:**
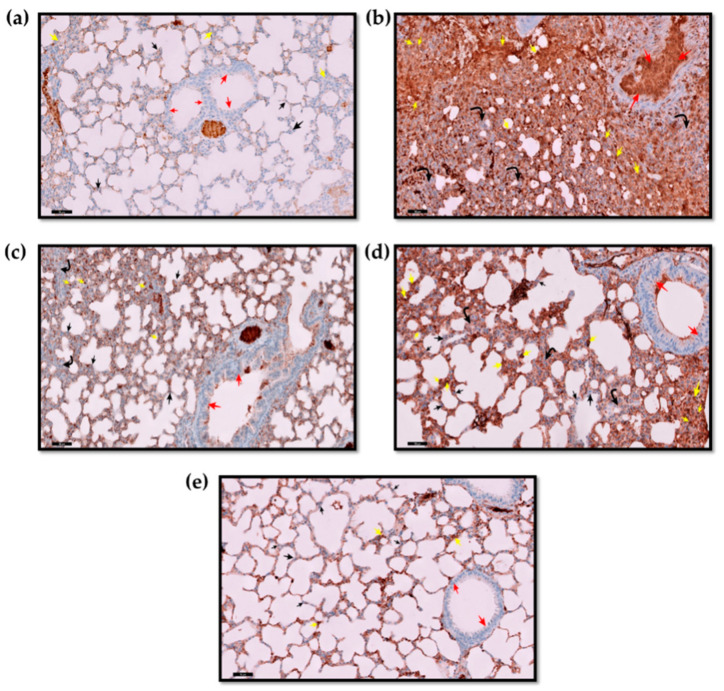
Immunohistochemical analysis of p53 in lung sections of mice with acute lung injury after treatment with AZ-NLC (IT) (immunohistochemical stain p53 × 20). (**a**) Control (vehicle) group: showed no immune expression of p53 in the AECs lining the alveoli (black ↑), cells lining the bronchiole (red ↑), and the cells within the thin IAS (yellow ↑). Notice non-specific positive reactions within septal blood vessels. (**b**) BLM group: showed marked strong positive immune expression of p53 (brownish colorations) in the nuclei of desquamated degenerated bronchiolar epithelium that filled the lumen (red ↑). Notice also that most alveoli were closed with marked aggregation of positive immune expression of p53 (yellow ↑) in the inflammatory cells in the thickened IAS (black curved arrow). (**c**) BLM + AZ (IT): showed less closed alveoli with a marked decrease in immune expression of p53 in the cells in the interstitium (yellow ↑), the AECs lining the alveoli (black ↑), and cells lining the bronchiole (red ↑). (**d**) BLM + AZ (PO): showed less decrease in immune expression of p35 in most of the AECs lining the alveoli (black ↑) and cells lining the bronchiole (red ↑). Notice that there was still moderate, strong positive immune expression of p35 (yellow ↑) seen in the thickened IAS and the interstitium (curved black arrow). (**e**) BLM + DEXA (IP): showed a marked decrease or negative in the immune expression of p53 in the AECs lining the alveoli (black ↑) and cells lining the bronchiole (red ↑). Notice a marked decrease or mild positive immune expression of p53 in the cells within the thin IAS (yellow ↑).

Figure 4 shows an immunohistochemical analysis of p53 in the lung sections of mice with acute lung injury after treatment with AZ-NLC (IT). Animals receiving vehicle (nanoformulation without drug) revealed no immune expression of p53 in the nuclei of alveolar epithelial cells (AECs), bronchiolar epithelium cells (BECs), or cells of the interalveolar septa (IAS). A moderate reaction was observed within blood vessels. However, animals treated with BLM (acute lung injury model) showed strong positive immune expression of p53 (as brownish color) and marked immune expression of p53 in the interstitial inflammatory cells within the thickened IAS. Different treatments modulated the expression of p53 in the lung tissues of the BLM-induced acute lung injury model.

**Figure 5:**
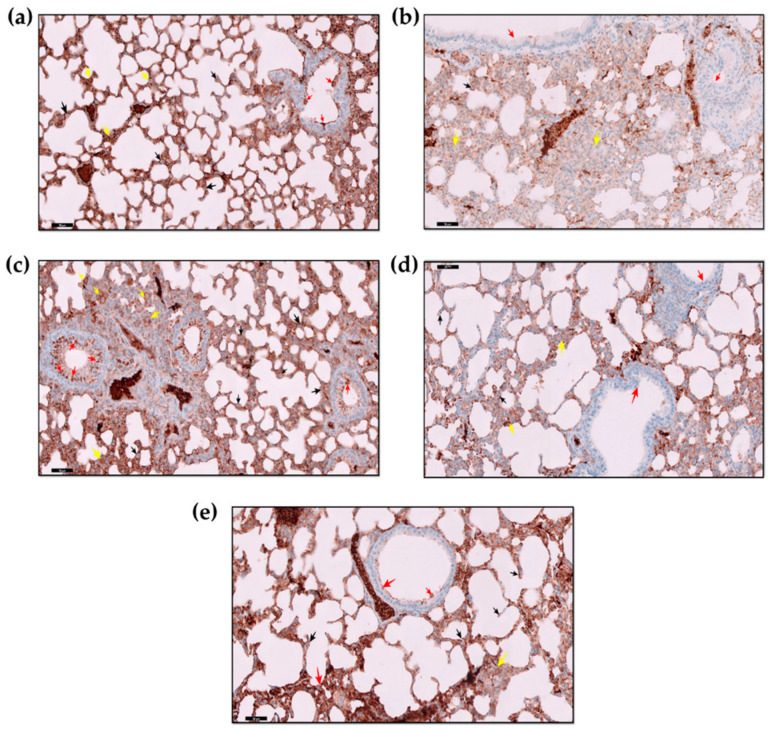
Immunohistochemical assessment of Bcl-2 expression in mice lungs of AZ nanoformulation compared with different groups (immunohistochemical stain BCL-2 × 20). (**a**) Control (vehicle): showing marked strong positive Bcl-2 immuno-expression in the cytoplasm of the AECs (black ↑) and cells lining the bronchiole (red ↑). Notice a few scattered strong positive cl-2 immuno-expression cytoplasmic reactions (yellow ↑) in the thin IAS cells. (**b**) BLM: showing negative Bcl-2 immuno-expression in bronchiolar epithelium cytoplasm (red ↑), mild positive Bcl-2 immuno-expression in a few AECs (black ↑), with negative Bcl-2 immuno-expression (yellow ↑) in cells of the kind IAS and interstitium. (**c**) BLM + AZ (IT): showing marked strong positive BCl-2 immuno-expression in the cytoplasm of the cells lining the alveoli AECs (black ↑) and in the cells lining the bronchiole (red ↑). Notice a moderate, strong positive cl-2 immuno-expression in the cells of the IAS and interstitium. (yellow ↑). (**d**) BLM + AZ (PO): showing strong positive Bcl-2 immuno-expression in the IAS cells (yellow ↑) and the AECs (black ↑). Notice the negative Bcl-2 immuno-expression in cells lining the bronchiole (red ↑) is seen. (**e**) BLM + DEXA (IP): showing marked strong Bcl-2 immuno-expression (yellow ↑) in the cells of the IAS ad interstitium. Notice an apparent moderate increase in Bcl-2 immuno-expression in the cells lining the bronchiole (red ↑) and in the AECs (black ↑).

Figure 5 shows the immunohistochemical assessment of Bcl-2 in mice lung tissues: the animal lung tissue of the control (vehicle) “nanoformulation without drug” exhibited evident positive Bcl-2 immune expression in the cytoplasm of AECs and BECs. However, in the IAS, there were a few scattered cells that showed positive Bcl-2 immune expression. On the other hand, BLM-treated mice showed negative Bcl-2 immune expression in BECs as well as interstitial cells, and a few foci of AECs that line the alveoli showed positive Bcl-2 immune expression. In the animals receiving IT AZ-NLC, a positive and strong cytoplasmic Bcl-2 immune expression was observed in the epithelium lining the bronchiole and the AECs that lined the alveoli, which showed moderately positive immune reactions as well as IAS interstitial cells. The lungs of mice receiving oral administration of AZ showed few cells with a mildly strong positive Bcl-2 immune expression found in the IAS and cells lining AECs. Notice the negative immune reactions in the cells lining the bronchiole. On the other hand, animals receiving IP DEXA showed marked strong positive Bcl-2 immune expression of IAS cells. Notice the moderate increase in the positive reaction of the cytoplasm of bronchiolar cells and the AECs.

## 3. Discussion

AZ is a macrolide antibiotic with a potential role in the management of COVID-19 as well as other pulmonary chronic diseases due to its immunomodulatory, anti-inflammatory, and antiviral activities [10,11,12,13,28]. However, concern was raised about its cardiac toxicity, especially when combined with other QT-prolonging drugs such as HCQ [14,15,16,17]. The low efficacy and serious side effects associated with the conventional dosage forms of AZ limit its use for the treatment of COVID-19. The inhalation route of drugs is optimal for the management of respiratory diseases including COVID-19 given the following considerations. The infection by SARS-CoV-2 causes a clinical syndrome that predominantly manifests initially as pneumonia [29]. Human lung tissues are extremely vascular and have a high surface area. These features enhance the bioavailability of inhaled drugs and allow a fast effect. Therefore, the inhalation route of drugs is optimal for the management of respiratory diseases, such as infections [22]. Some antiviral drugs against respiratory viral infections, e.g., influenza and respiratory syncytial virus were formulated as lung delivery systems [30,31].

NLC drug delivery systems provide a potent, customizable approach to selectively target pulmonary tissues. NLC has numerous benefits, such as a higher encapsulation efficiency (%EE), improved stability, enhanced biocompatibility, bioavailability, simplicity of manufacture, non-toxicity, and increased drug loading [27,32].

Thus, based on the aforementioned criteria, the current study aimed to design AZ nanoformulations suitable for inhalation. This nanoformulation was subjected to pharmaceutical characterization and preclinical efficacy investigations in a BLM animal model that likely has similar features to COVID-19.

In our study, the hot emulsification–ultrasonication method was used for the preparation of AZ-NLC [33]. Compritol^®^ 888ATO and almond oil were selected based on their proven success in the NLC formulation. Additionally, almond oil is a well-known natural oil with a good safety profile. Moreover, the used lipid substances were biodegradable and biocompatible. The used lipids have several advantages for the NLC formulation documented in the literature [32,34,35]. To promote the stability of the NLC, L-phosphatidyl choline was used as an amphiphilic surfactant. In addition, the combination of hydrophilic and lipophilic surfactants is documented to cause reduced particle sizes in comparison to using either alone [36]. After the preparation of AZ-NLC nanoformulations, the characterization of these formulations were tested, and all the following parameters were measured: the particle size, zeta potential, PDI, and in vitro release. The particle size is a pivotal parameter for nanocarriers as it affects their pharmacokinetics in the biological system. The average particle size of AZ-NLC was 453 nm, which indicates that the developed nanoformulations were within the nano-size range [37,38]. Zeta potential shows the surface charge of the nanoparticulate systems, which was −30.2 mV and refers to the stability of the formulation [39,40]. The PDI was 0.228, which refers to the reasonable distribution for the particle size of NLC within the nanoformulation [41,42]. In vitro release results of AZ-NLC exhibited initial enhanced drug release (49.57 ± 9.34) in the first two hours followed by a slower release pattern. It attained the following value (81.80 ± 8.17) after 24 h, which indicated a good release profile from the core of the NLC. The release pattern of drugs loaded in nanoformulations usually shows a biphasic pattern, which indicates homogenous dispersion. However, a very fast drug release may indicate drugs are entrapped on the surface; on the other hand, a very slow release suggests the release of drugs from the core of the nanoparticles [43]. A sustained release pattern of AZ from the lipid liquid crystalline nanoparticles was reported. These formulations provided better efficacy in the management of periodontitis [44]. Given these findings, we speculated that the current AZ formulation could allow a prolonged effect in lung tissues with once-daily dosing

In our study, the probable efficacy of the designed AZ-NLC was examined against an acute lung injury mouse model. This study used a BLM as a mouse model, a commonly utilized model for studying the impact of medications on the treatments of lung disorders [45]. Even though there is no ideal animal model for COVID-19, the BLM-induced model represents a well-established model of lung inflammation with pathological alterations (endothelins, epithelial cytopathy, surfactant loss, inflammatory infiltrates) similar to that observed with COVID-19 [46,47]. In this study, we administered the IT single dose of BLM (2.5 mg/kg), which resulted in the development of acute lung injury [48,49].

BLM induces pulmonary injury via oxidant injury and DNA strand damage. The inflammatory response is amplified in the early stages of BLM-induced lung damage, with an increase in inflammatory cytokines and monocyte recruitment [50,51]. Extreme cytokine production has been shown to mediate the etiology of acute lung injuries. Structural pulmonary injury is followed by these inflammatory cytokines induced by BLM [52,53]. TNF-α induces inflammation causing tissue damage and recruits neutrophils to the site of injury [52]. Excessive NF-kB stimulation plays a fundamental role in the inflammatory response via its capability to activate the release of several pro-inflammatory cytokines that may lead to inflammatory diseases, such as ulcerative colitis, asthma, and acute lung injury [52,54,55,56,57].

IL-6 is a pro-inflammatory cytokine that has a role in a variety of inflammatory processes. Its overexpression causes lung illnesses such as ARDS, pulmonary fibrosis, and chronic obstructive pulmonary disease. It may also have a role in the development of acute lung injury and fibrotic changes induced by BLM [58,59].

IL-1β is also a pro-inflammatory cytokine that results in cellular damage. Its activation exacerbates inflammation resulting in epithelial abnormalities within lung tissues [52].

Immunomodulatory and anti-inflammatory effects have been reported for AZ including the inhibition of pro-inflammatory cytokines production, alterations in autophagy, reducing neutrophil influx, and the induction of regulatory functions of macrophages. In lung tissue, AZ could also have antifibrotic and proapoptotic activities on primary fibroblasts that may suppress lung fibrosis [60,61,62,63].

In this study, the levels of the pro-inflammatory markers (IL-6, IL-1β, TNF-α, and NF-kB) were high in the pulmonary tissues of the BLM-induced model of acute lung injury. The administration of AZ-NLC IT at a low dose of 11 mg/kg, markedly alleviated the BLM-induced increase in the above-mentioned pro-inflammatory cytokines. This outcome is similar to the changes detected after oral AZ at a high dose of 88 mg/kg. The amelioration effects of AZ on pro-inflammatory markers were comparable to what was documented by other researchers [60,61,64,65,66,67,68]. These findings demonstrate the role of the inhalation route of administration in the management of lung diseases and its utility for local drug delivery to pulmonary tissues, the rapid onset of action, and high bioavailability [22,30,31,32].

In our study, the histological evaluation of the pulmonary tissues confirmed that a single dose of BLM resulted in acute lung injury in mice. These findings of histological changes induced by BLM have also been documented by several researchers [49,52,69]. All these histological alterations were attenuated by IT AZ-NLC nanoformulations at a relatively low dose compared to oral administration of AZ. The efficacy of AZ, HCQ chloroquine, and nilotinib to attenuate experimentally induced lung injury were reported [68,70,71]. The improved effect of the lung delivery of AZ-NLC nanoformulation was likely due to the local delivery of the drug to the site of injury and higher access of AZ to lung tissues. The advantages of this local delivery were also reported in many publications [22,30,31,32].

Apoptosis is a form of cell death that plays an important function to maintain cell homeostasis [72]. P53 is a cancer suppressor gene with a vital function in the governance of the cell cycle and apoptosis. Typically, in a normal cell, the level of the p53 protein is low, while the high level of the p53 protein may trigger DNA injury and other stress signals that have three major functions: DNA repair, growth arrest, and apoptosis [73,74,75]. In our study, the expression of p53 in lung tissues was studied. BLM induces a marked increase in the p53 in the lung alveolar and bronchial epithelium as well as thickened interalveolar septa. Similar findings were reported [76]. Treatments with AZ nanoformulations decreased p53 expression. The best results were observed after treatment with a low dose of AZ nanoformulations by IT, which provided an effect comparable to the IP treatment of DEXA. This effect of AZ on the p53 apoptotic marker was reported [77,78,79].

Bcl-2 is one of the most critical regulators of apoptosis [80]. It is an intracellular membrane-related protein, and its overexpression inhibits apoptosis [80,81]. It plays a paramount role in carcinogenesis, apoptosis, inflammation, fibrosis, and interstitial lung injury [82,83]. In the current study, the immune expression of the anti-apoptotic marker Bcl-2 was studied. A marked decrease in Bcl-2 was observed in the BLM-treated group in the lung alveolar and bronchial epithelium, as well as interalveolar septa, compared to the control. Treatments with AZ-ameliorated BLM induced a decrease in Bcl-2 expression. The improvement was seen after the IT administration of nanoformulations that nearly matched that provided with IP DEXA.

## 4. Materials and Methods

### 4.1. Materials

AZ and sweet almond oil were purchased from Sigma-Aldrich Co. (St. Louis, MO, USA). Compritol^®^ 888 ATO and Gelucire^®^ 44/14 were purchased from Gattefossé (Saint-Priest, France). L-phosphatidylcholine was purchased from Avanti Polar Lipids (Birmingham, UK). DEXA (4 mg/mL injection) was purchased from Amryia Pharmaceutical Industries, (Alexandria, Egypt).AZ (200 mg/5 mL) suspension was purchased from Arab Pharmaceutical Manufacturing Co. Sult-Jordan. Bleomycin sulfate 15 units vial was purchased from Korea United Pharm. Inc. The Enzyme-Linked Immunosorbent Assay (ELISA) kits for the inflammatory markers were purchased from My-bio-source Inc., (San Diego, CA, USA) and for protein, analysis were purchased from Abicam (Cambridge, UK).

### 4.2. Preparation of AZ-NLC

Synthesis of AZ-NLC was carried out following the hot emulsification–ultrasonication method. A detailed protocol is described in our previous publication [84]. Briefly, the lipid phase (10% *w*/*v*) is composed of solid lipid (Compritol 888 ATO^®^) 0.9 g, liquid lipid (sweet almond oil, 0.2 g), and phosphatidylcholine, L (amphiphilic emulsifier 2% *w*/*w* of lipid components) was mixed with AZ (50 mg) at 70 °C and then mixed with aqueous Gelucire^®^ 44/14 (1.5% *w*/*v*) while stirring at the same temperature. The dispersion was ultrasonicated using VCX750 (Sonics & Materials Inc., CT, USA) then cooled and stored at 4 °C.

### 4.3. Characterization of AZ-NLC

#### 4.3.1. Particle Size, PDI, and Zeta Potential

Particle size, polydispersity index (PDI), and zeta potential of the AZ-NLC formulations were performed by using a Malvern size analyzer (Nano ZSP, Malvern, UK). Samples were diluted in distilled water (1:20 *w*/*v* NLC–water ratio) for an optimum count of 50–200 kilo-counts per second (kcps) before measuring the size and estimation of PDI. For zeta potential measurement, the sample was measured undiluted. The mean of three measurements was presented.

#### 4.3.2. In Vitro Drug Release Evaluation

The in vitro release study of AZ from its NLC formulations was carried out by using the dialysis bag technique. The specified amount (1 mL equivalent to 5 mg) of prepared AZ-NLC formulations was placed in the dialysis bag (14 kDa molecular weight cut-off (MWCO)), then immersed in the 500 mL distilled water as dissolution medium (USP Dissolution Tester, apparatus II; Erweka, Germany). The dissolution medium was maintained at 37 °C and the rotation speed of the paddle was set to 75 rpm. Aliquots of 5 mL were taken from the dissolution medium after 0.5, 1, 2, 4, 6, 8, 12, and 24 h. Three samples were tested to determine the concentrations of AZ by the high-performance liquid chromatography (HPLC) method [85]. AZ concentrations were determined by the HPLC method, validated, and developed in our labs in terms of accuracy, linearity, and precision. Analysis was conducted using Agilent 1200 system with a diode array detector, Zorbax Extend C18 column (4.6 × 150 mm, 5 μm), a quaternary pump, and an auto-sampler (Palo Alto, CA, USA). The system was controlled by ChemStation software (Rev. B.01.03 SR2 (204). Isocratic elution at a 0.6 mL/min flow rate was utilized. The mobile phase was composed of acetonitrile/water in 0.1% formic acid (9:1). The wavelength was set at 210 nm. The AZ level in the injected samples was measured concerning the calibration curves.

### 4.4. In Vivo Experiments

#### 4.4.1. Animals and the Experimental Study Design

The experimental protocol was approved by the ethical committee of the faculty of Pharmacy at King Abdulaziz University with approval no. “PH-1442-71”. Twenty-five male, 90-day-old mice, weighing between 20 and 27 gm were used in this study. The mice were acclimatized for 7 days before the study and housed in groups in plastic cages (five mice each) at a room temperature adjusted at 22–24 °C with a 12-hourly diurnal cycle and were allowed free access to standard mice food and water. 

**Figure 6:**
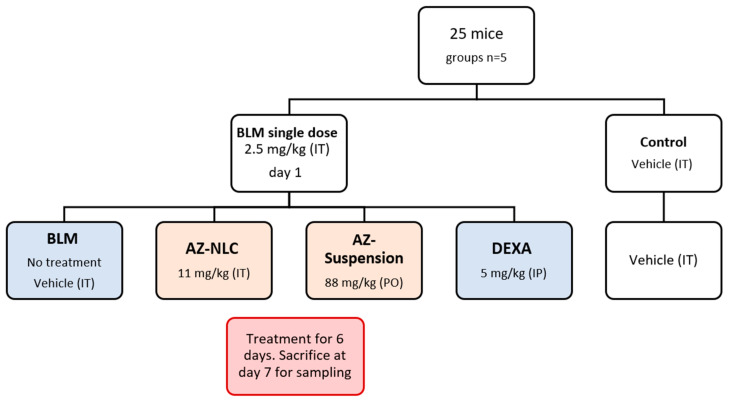
Flowchart of the experimental design. AZ: azithromycin; BLM: bleomycin; DEXA: dexamethasone; IT: intratracheal; PO: oral; IP: intraperitoneal; NLC: nanostructured lipid carriers.

The design is presented in Figure 6. A single dose of IT BLM (2.5 mg/kg) was administered for induction of acute lung injury [48]. The vehicle had the same composition as the nanoformulations but without a drug. The AZ dose was calculated based on the recommended human dose of SARS-CoV-2 [86,87]. For the IT dose of AZ-NLC, we considered the limited tolerability of mouse lungs to inhaled fluids and assumed increased bioavailability of medications given by inhalation in pulmonary tissues [88]. The dose of the positive control, DEXA, was selected based on the reported value [89]. Oral administration was conducted by gavage.

#### 4.4.2. Pro-Inflammatory Markers Analysis

Analysis of interleukin-6 (IL-6), tumor necrosis factor-α (TNF-α), nuclear factor-κB (NF-kB), and interleukin-1β (IL-1β) concentrations was quantified by ELISA by following manufacturer’s manual. The calibration and procedures were performed as instructed by the kit manufacturer. Measured pro-inflammatory cytokines in the tissues were expressed per *μg* protein. It must be noted that this experiment was performed concomitantly with a similar experiment presented in our previous publication [84], and under the same conditions.

#### 4.4.3. Preparation of Tissue Homogenate

A previously documented technique was used [23]. The lung tissues were washed with ice-cold phosphate buffer solution (PBS) 0.01 M, pH 7.4. Tissues weighing 100 mg were homogenized in 1 mL of cold PBS and promptly kept at −20 °C for 1 h. After that, they went through two freeze-thaw cycles before being centrifuged at the speed of 2000× *g* for 5 min at 8 °C using Fisherbrand accuSpin Micro 17/17R Microcentrifuge. The supernatant was collected and kept at −80 °C.

#### 4.4.4. Histopathological Evaluation

Lung tissue samples were fixed in a formalin solution (10%). Following dehydration, samples were cleared and embedded in paraffin [90]. A histologist sectioned 5-micron successive slices and stained them with hematoxylin and eosin for histological examination by Olympus light BX61 microscope. It must be noted that this experiment was performed concomitantly with a similar experiment presented in our previous publication [84], and under the same conditions.

#### 4.4.5. Immunohistochemical Evaluation

Paraffin sections of 5 µm thickness for the lung tissues were placed on positively charged slides; staining was undertaken using p53 stain as a marker of apoptosis [73,91] and Bcl-2 stain as a marker of anti-apoptotic effect [92,93]. The staining was performed following the protocol provided by the kit manufacturer.

### 4.5. Statistical Analysis

The program Statistical Package for the Social Sciences (SPSS, version 23.0) was used. A one-way analysis of variance (ANOVA) was adopted to make multiple comparisons, followed by Tukey’s post hoc test. Significance was defined as a *p*-value of less than 0.05. The mean (M) and standard deviation (SD) were used to show all data. GraphPad Prism software, version 5 (GraphPad^®^ Inc., San Diego, CA, USA) was used to create the graphs.

## 5. Conclusions

AZ-NLC pulmonary drug delivery systems were formulated successfully by using the hot emulsification–ultrasonication method. It showed acceptable pharmaceutical characteristics of particle size and release pattern. The pulmonary administration of AZ-NLC allowed for adequate AZ targeting in lung tissues. It reduced the inflammation and histological damage caused by bleomycin in lung tissues, most likely by downregulating p53 and modulating Bcl-2 expression. The current study represents a basis for further studies, such as formulation stability and pharmacokinetics, and further studies of lung toxicity with use for longer periods. Additionally, the inhalation route of administration is suitable for inpatients as well as outpatients.

## Data Availability

The data presented in this study are available on request from the corresponding author.
